# Development of 3D Printed Mitral Valve Constructs for Transcatheter Device Modeling of Tissue and Device Deformation

**DOI:** 10.1007/s10439-022-02927-y

**Published:** 2022-02-26

**Authors:** Marija Vukicevic, Shail Maharshi Mehta, K. Jane Grande-Allen, Stephen H. Little

**Affiliations:** 1 The Methodist DeBakey Heart & Vascular Center, 6550 Fannin Street, Suite 1851, Houston, TX 77030 USA; 2 Department of Bioengineering, Rice University, 6566 Main St., Houston, TX 77030 USA

**Keywords:** Mechanical testing, 3D printing, Mitral valve, 3D print materials, Transcatheter device modeling

## Abstract

Transcatheter mitral valve repair (TMVR) therapies offer a minimally invasive alternative to surgical mitral valve (MV) repair for patients with prohibitive surgical risks. Pre-procedural planning and associated medical device modeling is primarily performed in silico, which does not account for the physical interactions between the implanted TMVR device and surrounding tissue and may result in poor outcomes. We developed 3D printed tissue mimics for modeling TMVR therapies. Structural properties of the mitral annuli, leaflets, and chordae were replicated from multi-material blends. Uniaxial tensile testing was performed on the resulting composites and their mechanical properties were compared to those of their target native components. Mimics of the MV annulus printed in homogeneous strips approximated the tangent moduli of the native mitral annulus at 2% and 6% strain. Mimics of the valve leaflets printed in layers of different stiffnesses approximated the force–strain and stress–strain behavior of native MV leaflets. Finally, mimics of the chordae printed as reinforced cylinders approximated the force–strain and stress–strain behavior of native chordae. We demonstrated that multi-material 3D printing is a viable approach to the development of tissue phantoms, and that printed patient-specific geometries can approximate the local deformation force which may act upon devices used for TMVR therapies.

## Introduction

**Figure 1:**
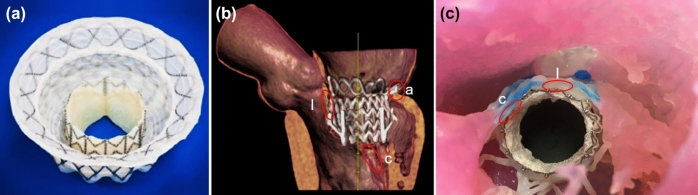
Bidirectional deformation of cardiac tissue and device. (**a**) Nitinol transcatheter mitral valve replacement (TMVR) device (Intrepid, Medtronic, USA); (**b**) Computed tomography image of the TMVR device implanted into a 3D printed mitral valve model incorporating tissue-specific material properties. (**c**) View from the left ventricle of the TMVR device deployed within a 3D printed mitral valve model. Regions of device and tissue deformation of the annulus (a), leaflet (l), and chordae tendineae (c) are identified (red ovals).

Mitral valve diseases are among the most prevalent heart diseases, and their prevalence increases with age.^16^ One of the most common defects of the mitral valve is mitral valve regurgitation (MR), affecting 7 million patients in the US annually.^10^ The traditional treatments for MR are surgical repair or replacement of the mitral valve (MV). However, as many as 49% of patients cannot undergo surgical treatment for severe degenerative MR due to advanced age, multiple comorbidities, and high surgical risks.^23^ There are several novel minimally-invasive therapies for mitral valve repair and replacement targeted to such patients.^9^ Pre-procedural planning of procedures such as transcatheter mitral valve repair (TMVR) is primarily based on digital anatomic models created from clinical imaging data. However, such digital-only modeling fails to incorporate the bidirectional deformation that occurs between the repair device and local mitral valve tissue as shown in Fig. 1. For example, when a nitinol TMVR (see Fig. 1a) device is deployed within a native mitral valve there are several deformation events that are expected: (i) the native mitral annulus is forced into a more circular configuration by the device; and the fully expanded device is compressed by the native annulus. This is an important aspect of device sizing since anchoring of the device often requires a 10–15% oversizing against the native annulus, thus creating consistent radial tension by the nitinol frame. (ii) The TMVR device necessarily displaces both anterior and posterior mitral leaflets into a vertical position within the LV (see Figs. 1b and 1c). The displaced anterior leaflet can create significant LVOT obstruction, however the magnitude of anterior leaflet displacement is constrained by its attachment to the chordae tendineae. As such, the sub-valvular apparatus may constrain the TMVR device deployment within the LVOT region or influence the final alignment of the entire device frame. Thus, there is need to develop patient-specific physical models to better inform patient and device selection protocols. The consideration of these deformation outcomes could influence the clinical decisions about type of TMVR device (e.g. self-expanding nitinol frame vs balloon-expandable cobalt-chromium frame); the size of the device for a specific patient; or even the decision to proceed with high risk surgery to avoid a predicted poor outcome with TMVR.

3D printing methods that replicate tissue-like mechanical properties could reveal important anatomic configurations that better predict patient outcomes from TMVR therapy, and may become an important tool for patient and device selection. Initially, 3D printed medical phantoms were fabricated predominantly of hard materials and used solely to demonstrate complex tissue geometry. The development of multi-material printers and flexible materials allowed for the development of flexible 3D printed phantoms for functional evaluation and medical device testing.^14^ Given the sensitivity of interactions, between mitral tissue and medical devices, i.e., 3D printed replicas should be fabricated using materials exhibiting mechanical properties similar to those of biological tissue. For example, since even minimal obstruction of the LVOT can have lethal consequences, 3D printed mitral leaflet constructs should replicate not only the geometry of the leaflet, but also the mechanical properties to allow replication of tissue deformation events during a TMVR procedure. However, data on the mechanical properties of 3D printed materials used for fabrication of cardiovascular models is limited.

The mitral valve apparatus is a complex and dynamic structure composed of the mitral annulus, anterior and posterior leaflets, chordae tendineae, and papillary muscles. The microstructure of the mitral valve elements, especially the leaflets and chordae, is responsible for effective function of the entire mitral apparatus. The mitral annulus is a collagenous structure, with the continuous anterior portion demonstrating much stiffer mechanical behavior than the non-continuous posterior annulus segments.^7, 1^ The inner structure of the mitral leaflets is organized in three different layers: the fibrosa facing the left ventricle, the spongiosa as the middle layer, and the atrialis on the atrial side. The layers each have different mechanical properties as they possess different amounts of elastin and load-bearing collagen fibers.^11^ In contrast, the chordae tendineae are comprised of an inner collagen core, a surrounding layer of elastic fibers, and an outer layer of endothelial cells.^15^

Few studies have investigated the mechanical performance of 3D printed materials used for the replication of cardiovascular tissue. With the recent development of new elastic photopolymers (such as Agilus and Rigur, Stratasys, Eden Prairie, Minnesota), there is a need for more specific testing and characterization of the mechanical properties of these photopolymers to determine their ability to replicate the mechanics of the mitral valve apparatus.

In this study, we developed novel 3D printed composites that approximated the mechanical properties of components of the mitral valve apparatus, including the mitral annulus, leaflets, and chordae. These composites were developed by replicating representative design elements of the native architecture of these components. Mechanical properties of these composites were investigated using uniaxial tensile testing and compared to those of the target biological tissue at physiologically relevant strains. Mechanical characterization of the 3D printed constructs involved measurements of ultimate stress and strain, elastic moduli, stress–strain relationships, and force–strain relationships.

## Materials and Methods

### Leaflet Design and Fabrication

To test and select the optimal combination of materials for each mitral valve element, we created different sample structures representative of the ultrastructure of the mitral annulus, leaflets, and chordae. All material samples were created using CAD-based software platforms 3-Matic (Materialize, Belgium) and Solidworks (Dassault Systems, Waltham, MA). Samples were fabricated using a PolyJet 3D printer (Object 500 Connex3, Stratasys, Eden Prairie, MN). Agilus and Rigur of different shore hardness were selected as the print materials. Printed materials with higher shore hardness values are generally associated with higher stiffness. Of the range of materials used in this study, the softest material was shore 27, while the stiffest material was shore 95.

### Mitral Annulus

Mitral annulus samples were designed as test coupons, or strips, that were 1 mm thick, 5 mm wide, and 20 mm long, and 3D printed using Agilus and Rigur material in a homogeneous configuration with a range of stiffnesses. The Annulus A1, Annulus A2, and Annulus A3 samples were 3D printed of Agilus shore 70, shore 85, and shore 95, respectively, whereas the Annulus B1, Annulus B2, and Annulus B3 samples were 3D printed from Rigur shore 70, shore 85, and shore 95, respectively.

### Mitral Leaflets

Due to the complexity and multi-layered ultrastructure of the mitral valve leaflets, we created both homogeneous and multi-material printed samples of mitral leaflets. The homogeneous ‘Leaflet A’ samples were fabricated of Agilus shore 27 (20 mm × 5 mm × 1 mm). The ‘Leaflet B’ samples were comprised of two 1 mm thick layers (20 mm × 5 mm × 2 mm), in which one layer was fabricated with Agilus shore 27 and the second layer with Agilus shore 50. The ‘Leaflet C’ samples were made of a 1 mm thick inner layer printed with Agilus shore 27, and a 0.4 mm thick outer shell printed of Agilus shore 70. Patient-specific mitral valve leaflets were reconstructed from computed tomography (CT) images of patient mitral valves using the imaging processing platform Mimics (Materialize, Belgium). The CT images were acquired using Siemens Force System with optimal contrast protocol. Image resolution was 512 × 512 pixels, with slice thickness of 0.6 mm.

**Figure 2:**
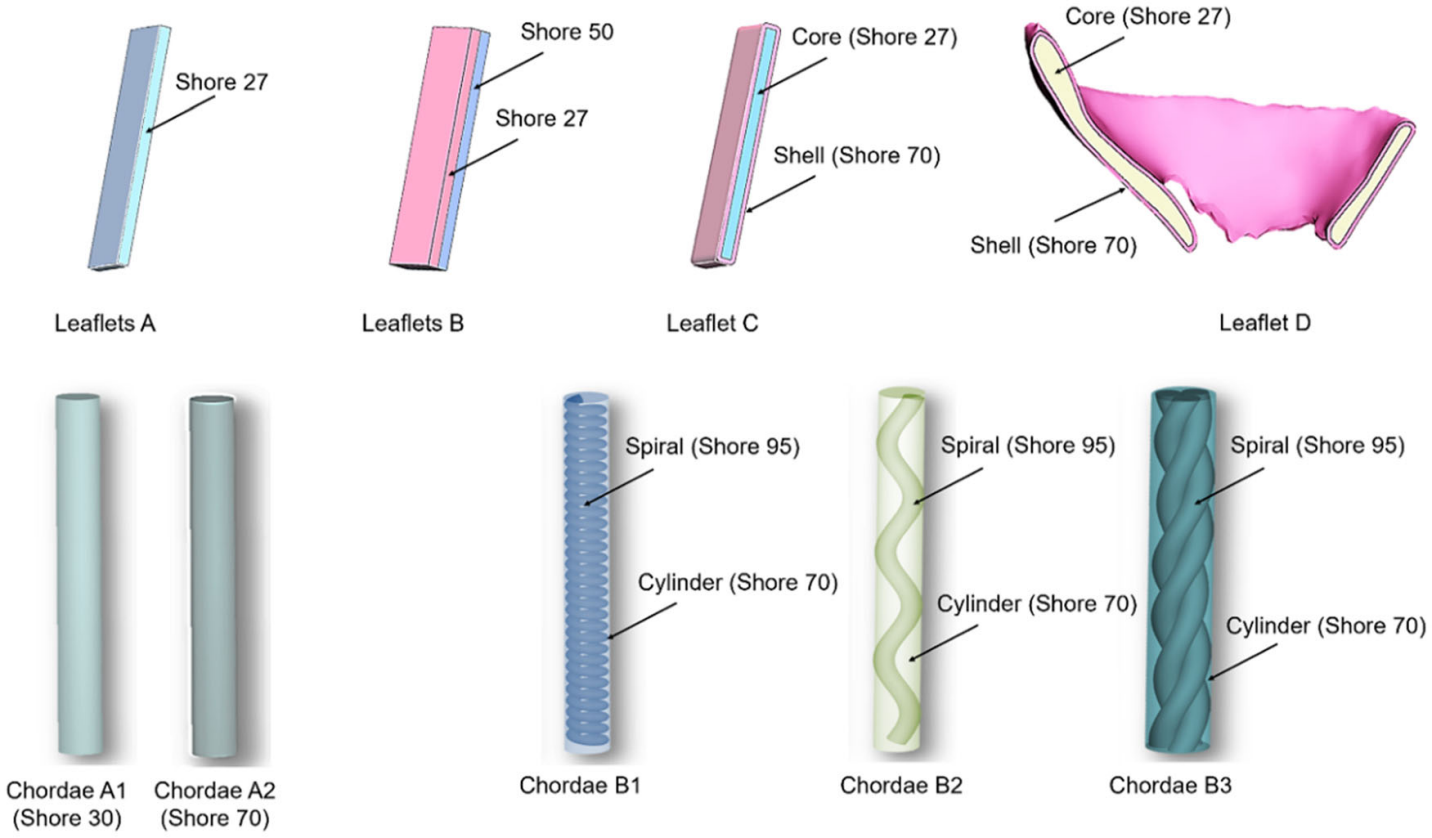
Design outline for 3D printed mitral valve constructs.

These leaflets, designated as sample group ‘Leaflet D’, were comprised of an inner layer printed using Agilus shore 27 and an outer shell printed using Agilus shore 70 and had a combined leaflet thickness that varied between 1 and 3 mm (as shown in Fig. 2). The patient-specific geometry of the leaflets and their irregular thickness was preserved.

### Mitral Chordae

To investigate the most appropriate printed material combination for the fabrication of chordae with native geometries reconstructed from patient CT scans, idealized chordal samples in the form of cylinders with fiber reinforcements were designed. A series of different fiber types were tested, including spiral, sinusoidal wave, and rope-like fibers. Reinforcement fibers and base materials were both printed to be 20 mm long. The spiral fiber reinforcement was 0.5 mm in diameter, whereas the number of revolutions per sample length was varied to influence their pitch length. After a series of iterative assessments that varied the reinforcement thickness and number of revolutions (illustrated in Appendix), we selected spiral reinforcements with 30 revolutions per 20 mm sample length for further study. The sinusoidal reinforcement fibers were designed using an equation-driven curve defined with the expression A sin (*ωt*), where *ω* represented angular frequency, A represented amplitude, and t represented the period. After another series of iterative assessments (data not shown), composite structures containing sinusoidal reinforcements with *ω* = 1, A = 0.5, and *t *= 0.4 were chosen for further study. To build on the fiber complexity, rope-like reinforcements were designed as three intertwined fibers of 1 mm thickness and 4 mm pitch. All fiber reinforcements were designed in Solidworks and incorporated into cylindrical chordal models using the CAD-based 3-Matic (Materialize, Belgium) platform. We also designed and tested samples of chordae without fiber reinforcements for reference.

The ‘Chordae A1’ and ‘Chordae A2’ sample groups were homogeneous cylindrical samples printed of Agilus shore 30 and shore 70, respectively. The ‘Chordae B1’ samples had spiral reinforcements, the ‘Chordae B2’ samples had sinusoidal reinforcements, and the ‘Chordae B3’ samples had rope-like reinforcements. The cylindrical base for all these three groups was printed of Agilus shore 70 and the reinforcement fibers were printed of Agilus shore 95. The printed annulus, leaflet, and chordae designs are summarized in Fig. 2.

### Selection of Relevant Physiological Strains

**Figure 3:**
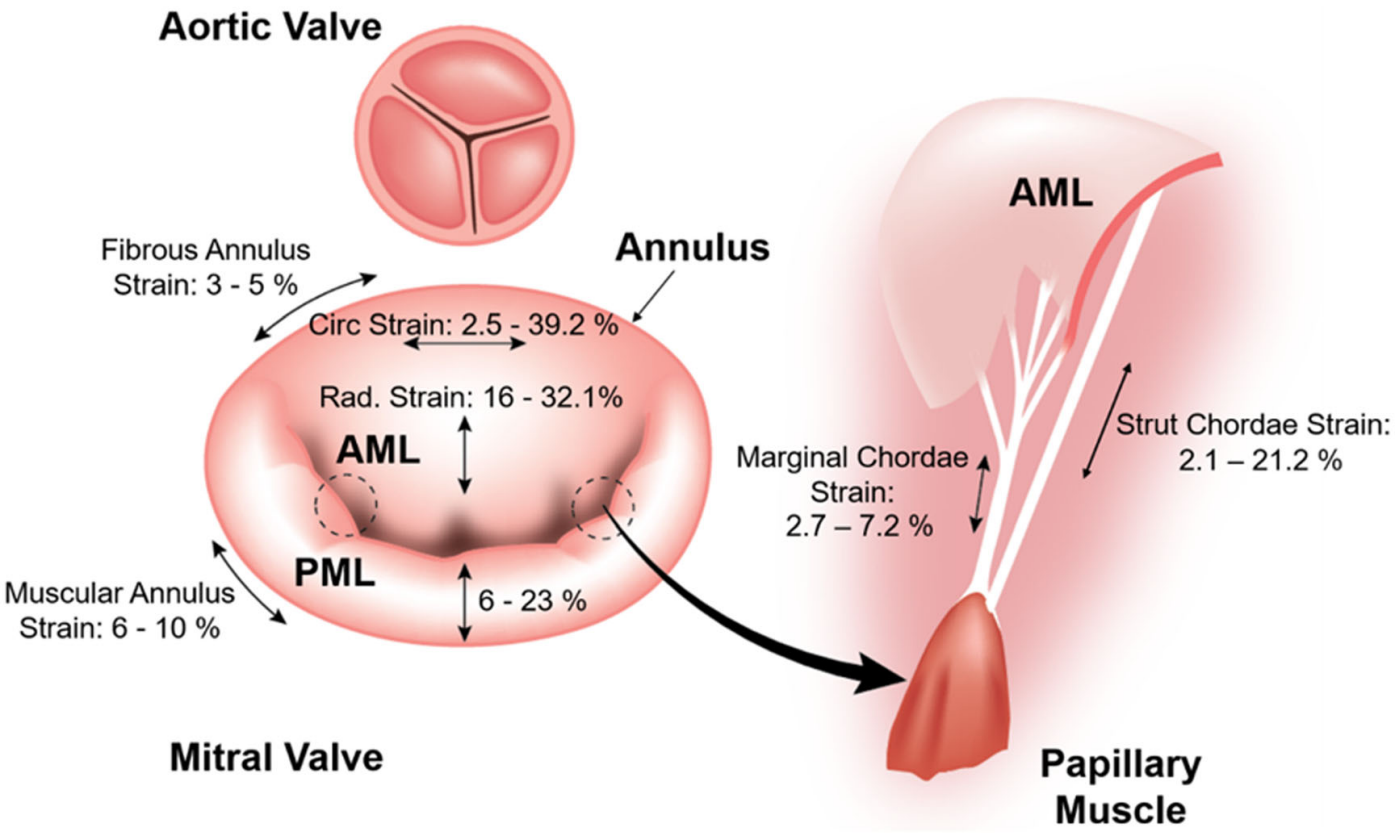
Range of reported physiological strains for the mitral valve apparatus were extracted from Gunning *et al*., Sacks *et al*., He *et al*., El-Tallawi *et al*., Grande-Allen *et al*., Liao *et al*. ^7^.

Different studies have found that the strain values of mitral tissue vary among mitral valve elements, and different ranges of physiological and maximal strain values have been reported. Based on a review of this literature, we analyzed the mechanical behavior of our printed constructs within physiologically relevant strains for each mitral valve element (Figure 3). Thus, we investigated the 3D printed annulus samples up to strain of 10%,^7^ and the 3D printed leaflet samples up to 25% strain. The strains experienced by chordae tendineae depend on their size and location. However, we were primarily interested in studying material behavior within physiologically averaged deformations. Therefore, we examined the mechanical behavior of chordae samples up to 10% strain.^12^

### Tensile Testing Procedure

Tensile testing was performed on printed model samples of the mitral valve annulus, leaflets, and chordae using a Bose Electroforce ELF 3200 Series III mechanical testing device as described previously.^22^ Samples were elongated either to failure or to the maximum instrument extension at a rate of 0.1 mm/s to apply a consistent strain rate across all printed samples. A subset of our samples was also tested at a 10x higher strain rate to quantify Agilus’ rate-dependent mechanical behavior.

In order to compare printed material behavior with that of biological tissue, tensile testing was also performed on chordae samples extracted from human tissue, as described previously.^5^ All human tissue samples used within this study were collected under an institutional review board approved protocol (IRB (3N) 0511-0100). The samples were obtained from recipient hearts of male patients (59–68 years old) that underwent heart transplantation. The tissue samples were obtained immediately after the transplantation and pathological inspection.

Chordae were preconditioned to 10% strain at 1 Hz for 20 cycles, and then were subjected to a pull-to-failure protocol at 0.1 mm/s. Testing was carried out in phosphate buffered saline solution (PBS) at 37 °C. Data from existing literature was used to make comparisons with printed material for mitral valve leaflets and annuli. Three replicates for each group were tested.

### Quantification of Mechanical Properties

Force-displacement data was converted to stress–strain data by dividing the force by the cross-sectional area perpendicular to the direction of testing to calculate stress, and by dividing the sample displacement by the sample gauge length to calculate engineering strain. Measurements of initial sample thickness and width (for cross-sectional area calculations) were obtained with ImageJ (NIH, Bethesda, MD) using images of samples taken via a stereomicroscope (Leica Microsystems, Buffalo Grove, MN).

Stress–strain and force–strain curves were generated up to the aforementioned strain levels for all printed components of the mitral valve apparatus. These curves were further analyzed to determine the mechanical behavior at specific strain magnitudes. For printed mitral valve annuli, we measured average force and average stress at 2 and 6% strain, in addition to tangent modulus at 2 and 6% strain. These values were selected to allow us to make comparisons with testing data for biological tissue in existing literature, as well as for their physiological relevance. The tangent modulus at a specific strain level was measured by first fitting a quadratic curve locally around the datapoint corresponding to that strain level, then finding the equation of the line tangent to that quadratic curve at that strain level. The slope of this tangent line was taken to be the tangent modulus at that strain level. For printed mitral valve leaflets, ultimate force, stress and strain were obtained, in addition to measurements of elastic modulus between 5 and 25% strain. Similar characterization was performed on mitral leaflet data with comparison to published literature.^22^ Such measurements were also obtained for printed and native chordae, where the elastic modulus was measured between 0 and 10% strain.

### Statistical Analysis

The average stress–strain curves for each individual group were generated by obtaining the stress value at strain values closest to a desired strain increment for a replicate in that group, then averaging stress values across all replicates for that group. This process was then repeated across all strain increments and groups to obtain averaged stress–strain curves. All data processing was performed using custom Python code,^17^ with the resulting values reported as mean ± SD. One-way ANOVAs were performed on tensile elastic modulus data from printed constructs and biological tissue. Post-hoc testing was performed using Tukey’s HSD test, and statistical significance was accepted for *p *values less than 0.05.

## Results

### Mechanical Testing of 3D Printed Mitral Annulus

**Figure 4:**
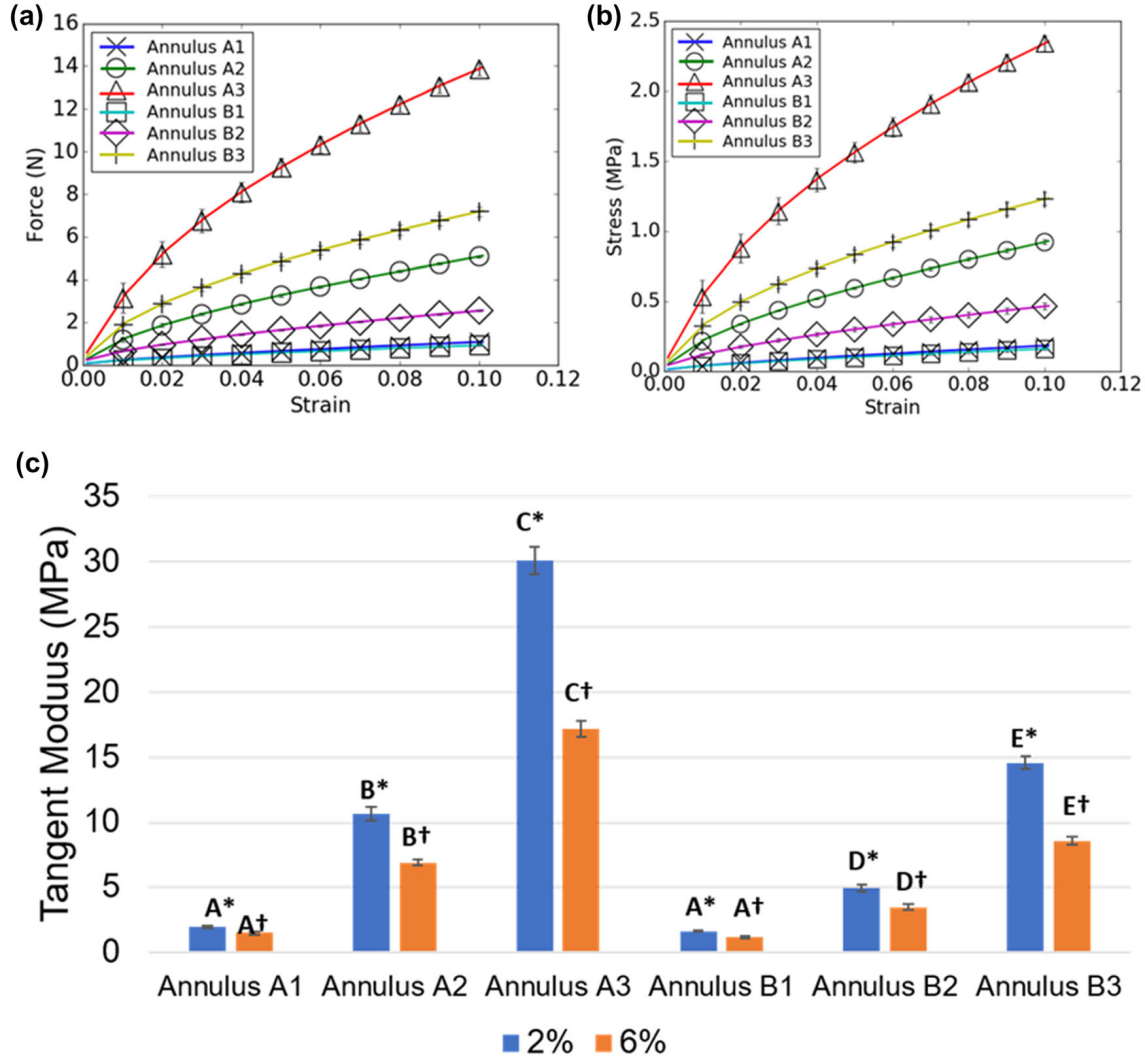
**a** Force–strain and **b** stress–strain curves for 3D printed mitral valve annulus samples for physiological strains. **c** Tangent Elastic moduli for 3D printed mitral valve annulus samples. Results are presented as mean ± SD. *N *= 3 for all indicated groups. The tangent moduli at different strains were not compared statistically. Comparisons between tangent moduli at 2% strain are indicated using *, and comparisons between tangent moduli at 6% strain are indicated using †. Groups sharing a letter are not significantly different from each other. *p < 0.05 and †*p *< 0.05 for all indicated groups.

After analyzing the force–strain and stress–strain behavior of Agilus and Rigur annulus sample, it was determined that the Annulus A3 and Annulus B3 samples demonstrated the highest forces under strain. The Agilus material samples (Annulus A1–A3) exhibited higher forces at all strain values as compared to the Rigur material samples (Annulus B1–B3, Fig 4a). These trends were preserved for the stress–strain curves as well (Fig. 4b). All 3D printed materials exhibited non-linear mechanical behavior, with initial high-stiffness behavior in the small strain regime (0–2%), followed by a region of lower stiffness at higher strains (2–10%). The force and stress values at 2 and 6% strain are presented in Table 1.

The tangent moduli of 3D printed samples of Agilus and Rigur at 2% and 6% strain were also determined (Fig. 4c and Table 1). Tangent moduli measured at 2% strain were higher than tangent moduli measured at 6% for all sample groups. Annulus material printed using a higher shore hardness demonstrated higher tangent moduli; however, material printed using Rigur material demonstrated lower tangent moduli than material printed using Agilus. All comparisons between Agilus and Rigur constructs for tangent moduli measured at the same strain levels were significantly different for all sample groups except for the comparison of Annulus A1 and B1. The overall performance of Agilus material was more similar to native annular tissue, therefore the following tests were performed on Agilus materials (Tables 2 and 3).

**Table 1:** Mechanical property measurements for printed mitral valve annulus constructs.

Material property	Shore hardness	At strain 2%			At strain 6%		
		Tangent modulus (2%) (MPa)	Stress (MPa)	Force (N)	Tangent modulus (6%) (MPa)	Stress (MPa)	Force (N)
Annulus A1	70	1.94 ± 0.09^a^	0.06 ± 0.00^a^	0.35 ± 0.02^a^	1.49 ± 0.11^a^	0.13 ± 0.00^a^	0.74 ± 0.02^a^
Annulus A2	85	10.66 ± 0.56^b^	0.34 ± 0.01^b^	1.85 ± 0.08^b^	6.92 ± 0.19^b^	0.66 ± 0.01^b^	3.66 ± 0.05^b^
Annulus A3	95	30.10 ± 1.02^c^	0.87 ± 0.10^c^	5.19 ± 0.60^c^	17.20 ± 0.62^c^	1.74 ± 0.07^c^	10.33 ± 0.39^c^
Annulus B1	70	1.65 ± 0.03^a^	0.05 ± 0.00^a^	0.32 ± 0.01^a^	1.18 ± 0.09^a^	0.11 ± 0.01^a^	0.64 ± 0.02^a^
Annulus B2	85	4.96 ± 0.24^d^	0.17 ± 0.01^a^	0.95 ± 0.01^a^	3.46 ± 0.22^d^	0.33 ± 0.02^d^	1.83 ± 0.04^d^
Annulus B3	95	14.57 ± 0.52^e^	0.49 ± 0.02^d^	2.87 ± 0.13^d^	8.58 ± 0.31^e^	0.92 ± 0.04^e^	5.37 ± 0.21^e^

Annulus A1, A2, and A3 were printed of Agilus material of shore 70, 85, and 95 hardness, respectively. Annulus B1, B2, and B3 were printed of Rigur material of shore 70, 85, and 95 hardness, respectively. Groups sharing a letter within the same column are not significantly different from each other (*p *< 0.05 for all indicated groups). Results are presented as mean ± SD. *N *= 3

**Table 2:** Mechanical property measurements for printed and biological mitral valve leaflet constructs.

Material property	Shore hardness	Elastic modulus (MPa)	Maximum observed strain (%)	Maximum observed force (N)	Maximum observed stress (MPa)	Failure achieved
Leaflets A	27	0.51 ± 0.01	58 ± 3	1.38 ± 0.12	0.30 ± 0.01	N
Leaflets B	27/40	0.63 ± 0.01	64 ± 3	4.25 ± 0.41	0.41 ± 0.01	N
Leaflets C	27/70	0.51 ± 0.05	58 ± 1	3.11 ± 0.33	0.31 ± 0.04	N
Leaflets D	27/70	0.95 ± 0.02	60 ± 8	7.07 ± 1.30	0.54 ± 0.11	Y
Mitral Anterior Leaflet	N/A	2.20 ± 0.55	49 ± 4	5.90 ± 1.60	0.96 ± 0.12	Y
Mitral Posterior Leaflet	N/A	1.74 ± 0.48	56 ± 6	3.74 ± 1.34	0.61 ± 0.16	Y

All printed leaflets were fabricated using Agilus. Leaflets A were printed with a homogeneous shore 27 formulation, Leaflets B were printed as bilayer constructs of shore 27 and shore 50 hardness, Leaflets C were printed with a shore 27 inner core and shore 70 outer shell, and Leaflets D were printed to patient-specific geometries with a shore 27 inner core and shore 70 outer shell. Results are presented as mean ± SD. *N  *= 3

**Table 3:** Mechanical property measurements for printed and biological mitral valve chordae constructs.

Material property	Shore hardness	Elastic modulus (MPa)	Maximum observed strain (%)	Maximum observed force (N)	Maximum observed stress (MPa)	Failure achieved
Chordae A1	30	0.52 ± 0.02	53 ± 5	1.13 ± 0.08	0.24 ± 0.02	N
Chordae A2	70	1.43 ± 0.09	49 ± 6	3.17 ± 0.29	0.66 ± 0.06	N
Chordae B1	70/95	3.50 ± 0.38	47 ± 7	6.18 ± 0.66	1.22 ± 0.18	Y
Chordae B2	70/95	2.80 ± 0.65	60 ± 17	6.45 ± 1.96	1.28 ± 0.48	Y
Chordae B3	70/95	7.23 ± 0.87	56 ± 2	12.86 ± 1.11	2.54 ± 0.20	Y
Mitral anterior chordae	N/A	31.88 ± 49.49	16 ± 7	1.73 ± 1.44	4.32 ± 4.55	Y
Mitral posterior chordae	N/A	62.64 ± 43.04	13 ± 2	1.50 ± 0.52	6.45 ± 4.24	Y

All printed chordae were fabricated using Agilus. Chordae A1 and A2 were printed using homogeneous formulations of shore 30 and shore 70 hardness, respectively. Chordae B1 were fabricated with spiral reinforcements, Chordae B2 were fabricated with sinusoidal reinforcements, and Chordae B3 were fabricated with rope-like reinforcements. Reinforcements were fabricated with shore 95 hardness, and base material was fabricated with shore 70 hardness. Results are presented as mean ± SD. *N *= 3

### Mechanical Testing of 3D Printed Mitral Valve Leaflets

**Figure 5:**
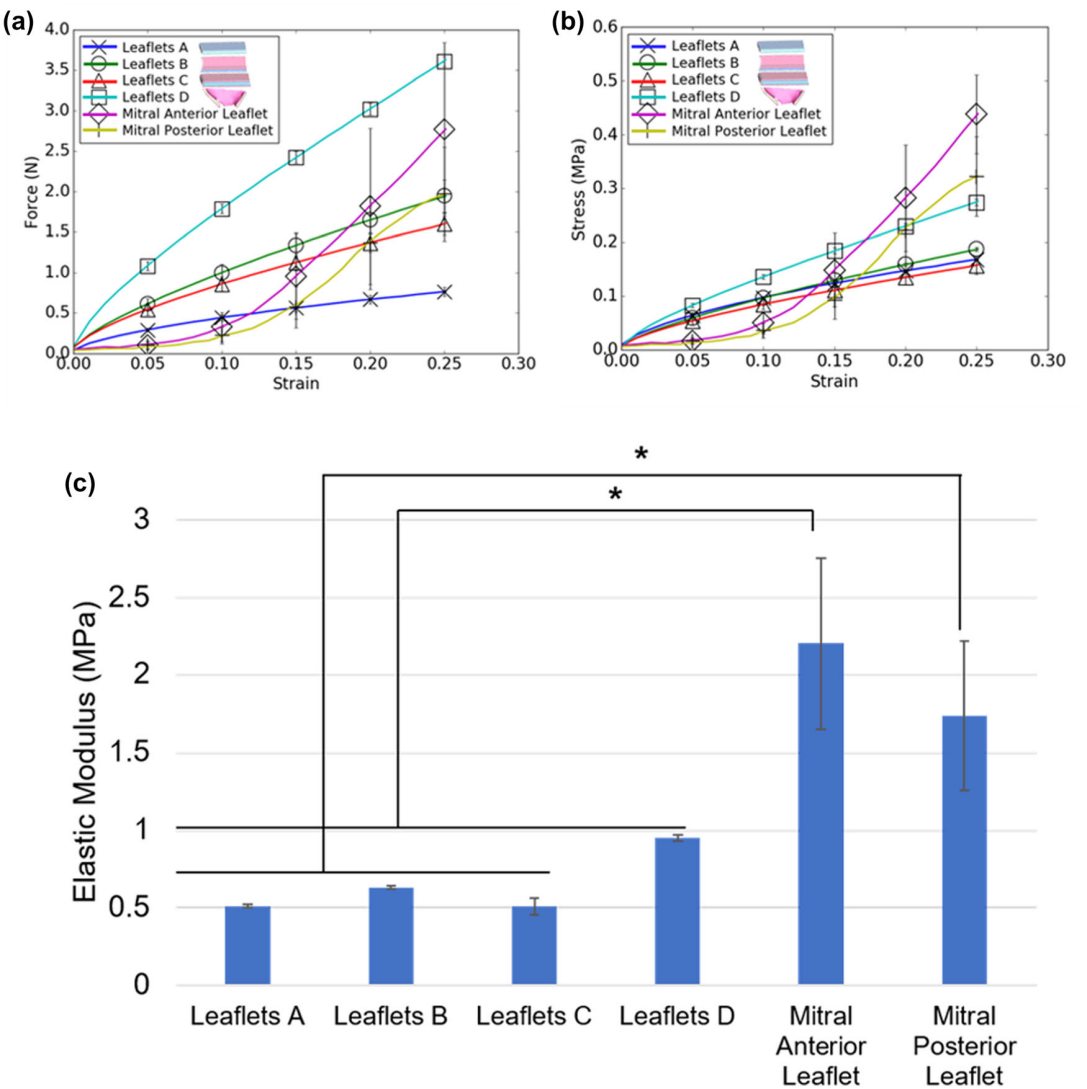
**a** Force–strain and **b** stress–strain curves for 3D printed mitral valve leaflet samples for physiological strains. **c** Elastic moduli for 3D printed mitral valve leaflet samples. Moduli were measured between 5 and 25% strain. Results are presented as mean ± SD. *N *= 3 and *p < 0.05 for all indicated groups. Model designs for composite printed structures are indicated on the figure legend.

Force–strain curves for both printed and biological mitral valve tissue were determined and depicted in Fig. 5a. For initial deformations (strains < 12%), elongation of the homogeneous Leaflet A samples resulted in forces that were similar to those of porcine anterior and posterior leaflets. At higher strain levels, the composite materials (Leaflet B, C, and D samples) exhibited force levels that were more similar to the biological leaflets (Fig. 5a).

Corresponding stress–strain curves for printed and biological mitral valve tissue were determined and plotted in Fig. 5b. The stress–strain behavior of printed materials was approximately similar to that of biological valves; however, in a manner similar to what was observed with the force–strain curves, the opposite concavity of the printed material curves resulted in slightly overshooting the stress–strain response at low strains and slightly undershooting the stress–strain response at high strains in comparison with biological leaflets. Leaflet A, B, and C samples showed a similar stress–strain response and matched biological stresses better at lower strains (< 15%), whereas Leaflet D samples indicated a better match at higher strains (Fig. 5b). When considering all sample groups, the printed materials were able to recapitulate the stresses displayed by biological samples between 12 and 20% strain.

The elastic modulus for each sample group was determined and plotted in Fig. 5C. Elastic modulus values, maximum observed stresses and forces, and maximum observed strain of all printed leaflet samples along with porcine anterior and posterior leaflet samples were presented in Table 2. There was no significant difference in elastic modulus among the printed materials. Elastic moduli for Leaflet A, B, and C samples were significantly lower than those of biological valves. However, the elastic moduli of Leaflet D and Mitral Posterior Leaflet samples were not statistically different from one another.

Ultimate strain measurements for all printed valves exceeded those of biological leaflets. The Leaflet A, B, and C samples did not undergo failure before the maximum extension limit of the tensile testing instrument was reached, indicating that the ultimate strains of these printed groups are even higher than indicated in Table 2. While the printed materials were unable to match the ultimate stress values of biological tissue, they were able to recapitulate the ultimate force values demonstrated by both anterior and posterior mitral valve tissue.

### Mechanical Testing of Biological and 3D Printed Mitral Valve Chordae

**Figure 6:**
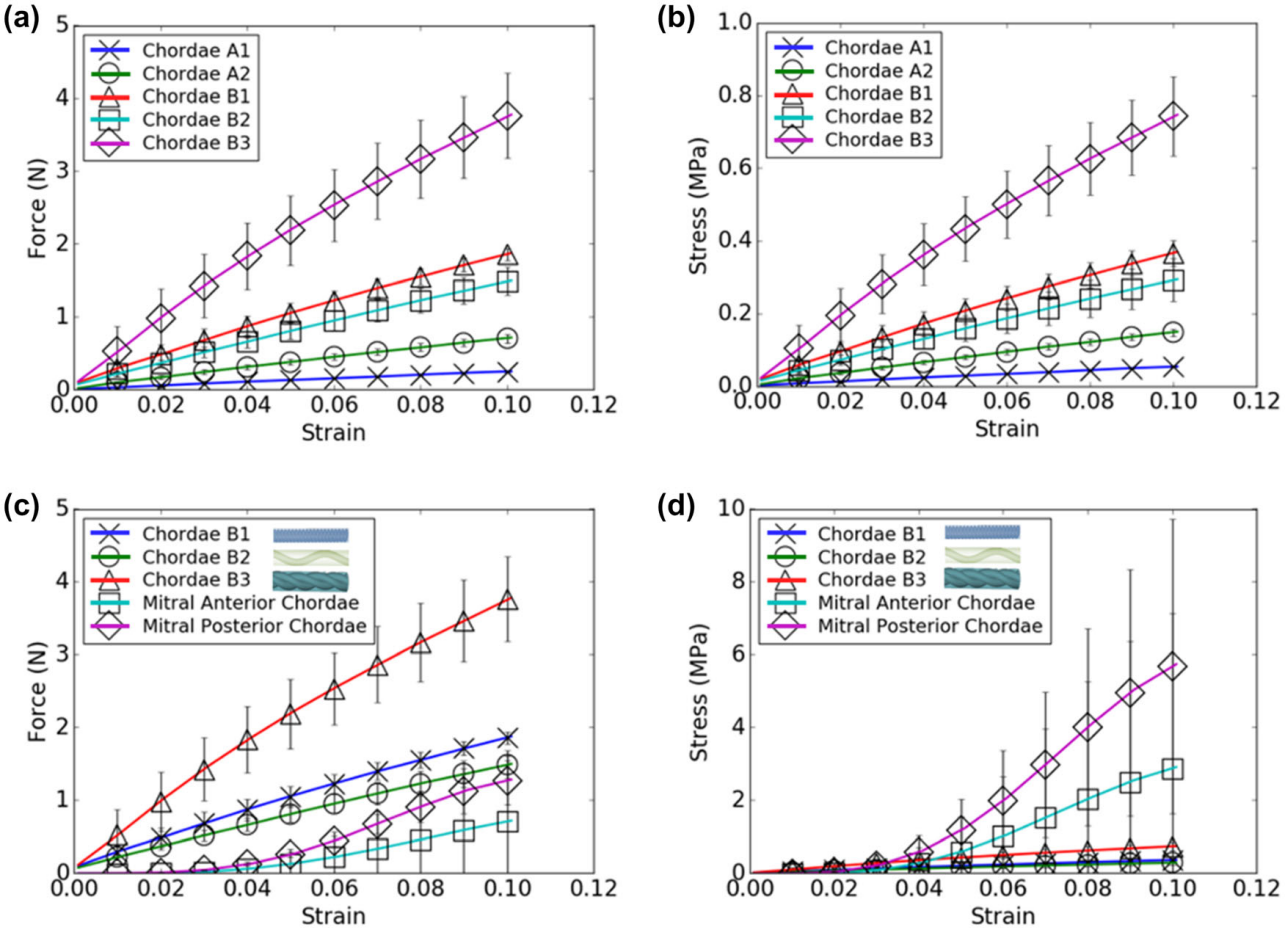
**a**, **c** Force–strain and **b**, **d** stress–strain curves for 3D printed mitral valve chordae samples for physiological strains. Results are presented as mean ± SD. Model designs for composite printed structures are indicated on the figure legend.

The force–strain and stress–strain behavior of printed and biological chordae samples was determined and plotted in Fig. 6, showing comparisons between all printed samples (Figs. 6a and 6b), and between printed and biological samples (Figs. 6c and 6d). The homogeneous chordae sample groups Chordae A1 (shore 30) and A2 (shore 70) showed a lower force and stress response compared to the groups Chordae B1, B2, and B3, which were comprised of a base material (shore 70) and stiffer reinforcement (shore 95). Relative trends between all printed chordae groups were preserved across both force–strain and stress–strain curves.

Force–strain curve comparisons between the composite printed models and biological chordae indicated that printed composite chordae showed comparatively higher force responses than biological chordae at all strain levels. Chordae models B1 and B2 showed the closest similarity in forces to biological chordae, but only at higher strain levels (8–10%). Conversely, stress–strain curve comparisons between the printed composite and biological chordae indicated that the printed chordae could approximately match the stresses shown by biological chordae at low strain levels (0–5%) but were unable to match their stress response at higher strains.

**Figure 7:**
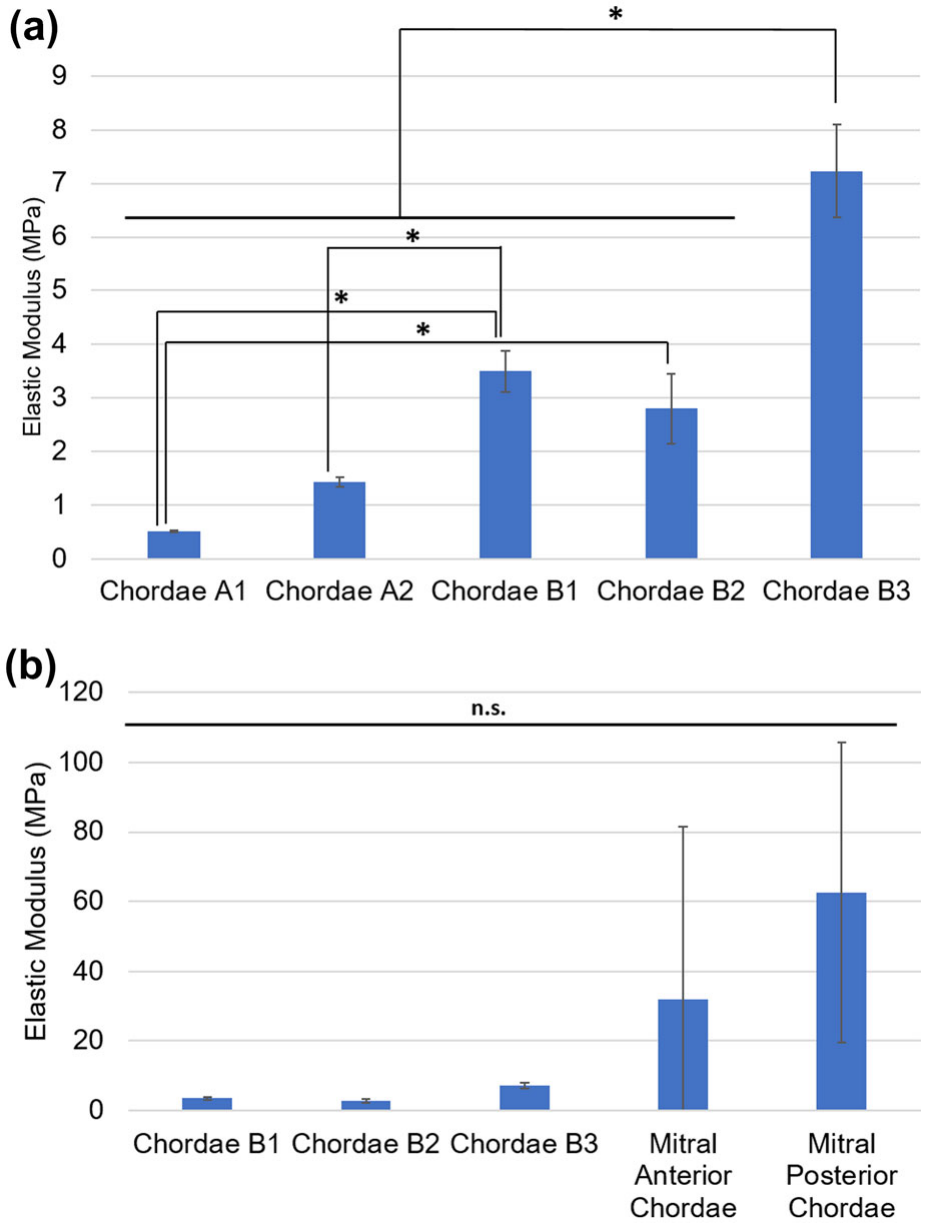
Elastic moduli for 3D printed mitral valve chordae samples. Moduli comparisons between (**a**) printed samples, and between (**b**) printed composite samples and biological samples. Moduli were measured between 0 and 10% strain. Results are presented as mean ± SD. *N *= 3 and *p < 0.05 for all indicated group.

Elastic moduli for printed and biological chordae as measured between 0 and 10% of their stress–strain curves are shown in Fig. 7. Between the printed material models, the sample group Chordae B3 showed the highest elastic modulus, and composite models reinforced with Shore 95 fibers generally indicated a higher elastic modulus than the homogeneous models A1 and A2. Statistically significant differences between printed groups are detailed in Fig. 7a. The elastic moduli of both anterior and posterior mitral valve chordae were up to an order of magnitude higher than these reinforced models; however, statistical analysis did not indicate a significant difference between printed models and biological chordae.

Ultimate strain, maximum force, and maximum stress data is displayed in Table 3. The ultimate strain behavior of printed chordae was far higher than that of biological chordae. The homogeneous chordae models A1 and A2 did not undergo failure prior to the maximum extension limit of the tensile testing instrument, indicating that their ultimate strains were higher than the maximum observed strain listed in Table 3. Generally, the printed materials displayed higher maximum forces and lower maximum stresses as compared to biological chordae. This observation is consistent with measurements of cross-sectional area of the printed and native chordae, where the cross-sectional area for printed reinforced chordae (Chordae B1, B2, and B3) was 5.11 ± 0.28 mm^2^, and that of the native chordae was 0.50 ± 0.47 mm^2^ (anterior) and 0.29 ± 0.15 mm^2^ (posterior).

### Mechanical Testing of 3D Printed Material at Different Strain Rates

**Figure 8:**
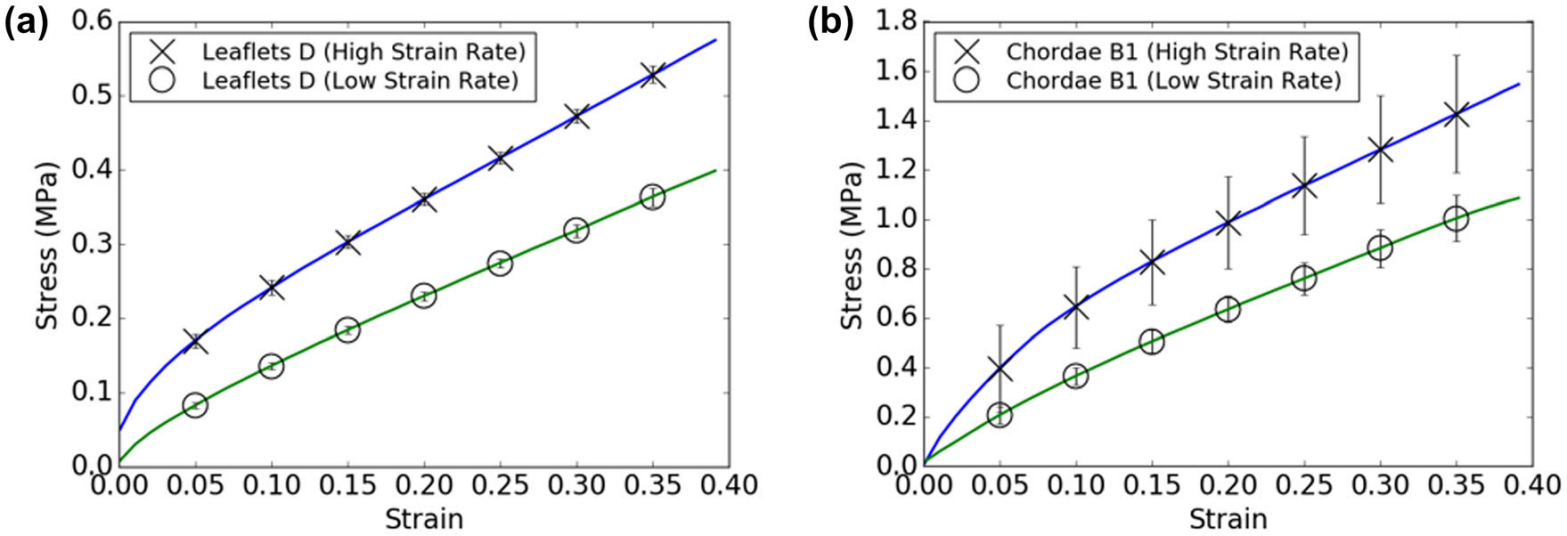
Stress–strain curves for 3D printed **a** patient-specific leaflets (Leaflets D) and **b** spiral-reinforced chordae (Chordae B1) at two different strain rates, 0.07/s (high strain rate) and 0.007/s (low strain rate). Results are presented as mean ± SD. *N* = 3 for all samples.

Samples printed using Agilus material were found to produce higher stresses at the same strains if tested at a higher strain rate (Fig. 8). Trends for increases in stress response with increase in testing rate were also preserved for sample force–strain curves (data not shown). The degree of increase in material stiffness with increasing strain rate seemed independent of printed material architecture, i.e., trends for increases in stress response with increase in testing rate were preserved between leaflets with a layered core-and-shell structure and chordae with embedded reinforcements of higher stiffness.

## Discussion

3D printing is becoming increasingly popular for the fabrication of patient-specific cardiovascular models for planning complex surgical procedures. However, most of these models are printed using materials with insufficiently characterized mechanical properties, resulting in a poor understanding of the similarity between printed patient-specific models and actual patient biological tissue. This study worked towards designing 3D printed constructs fabricated with the photopolymers Agilus and Rigur in a combination of different stiffnesses that best approximated the mechanical properties of tissue from components of the mitral valve apparatus. Inspiration from biological architecture was used to fabricate 3D printed mimics of mitral valve annuli, leaflets, and chordae. Uniaxial tensile testing was performed on the resulting constructs and the measured mechanical properties were compared to those of the target biological tissue.

The 3D-printed materials favorably replicated the material behavior of the mitral valve annulus at physiological strains. In vivo studies of ovine mitral valve annuli indicated a maximum physiological strain of 3–5% for the fibrous portion of the mitral annulus, and 6–10% for the muscular portion of the mitral annulus.^20^ Gunning and colleagues determined that the mitral annulus does not possess a homogeneous stiffness, and that the anterior portion of the annulus is much stiffer than the posterior portion. The mitral commissural and posterior annuli were found to have a median tangent modulus of between 1 and 3 MPa at 2% strain, and 1–2 MPa at 6% strain. The mitral anterior annuli, however, displayed a median tangent modulus of 28.15 MPa at 2% strain and 21.55 MPa at 6% strain.^7^ Comparisons with the stress–strain curves and tangent moduli reported in this study indicate that that the sample group ‘Annulus A3’ represents the optimal choice for printing the anterior portion of the annulus, while the sample groups ‘Annulus A1’ and ‘Annulus B1’ would be suitable for replication of the posterior and commissural annulus segments. It should be noted that Rigur samples demonstrated lower stiffnesses than Agilus samples at higher shore values, although the shape of the stress–strain and force–strain curves for each group were similar. This indicates that Agilus constructs should be used for mitral annulus sections requiring higher stiffnesses, and Rigur may be considered for mitral annulus sections requiring lower stiffnesses.

Direct comparisons with the study performed by Gunning and colleagues present a challenge as the study by Gunning utilized measurements of true stress and strain as opposed to measurements of engineering stress and strain; however, such comparisons may still work to inform broad design guidelines. The measured decrease in tangent modulus at 6% strain, compared to tangent modulus values at 2% strain for printed annuli reflects the nature of the stress–strain curve of the Agilus and Rigur photopolymers, which exhibit higher stiffness at low strain values followed by a region of lower stiffness. All printed materials examined in this study exhibited this form of mechanical nonlinearity.

3D printed mitral valve leaflet models were able to reproduce the magnitude of stresses and forces of native mitral leaflet tissue at physiological strains as well. Comprehensive understanding of the mechanical properties of mitral leaflets is crucial for the development of physiological patient-specific 3D printed models, as well as for the optimization and a design of novel catheter-based devices for mitral valve repair and replacement. This understanding could better inform clinical decision-making for determining medical device suitability and sizing in order to maximize beneficial patient outcomes. A series of studies have investigated and sought to characterize the mechanical parameters of mitral valve leaflets.^6^ These studies have all indicated that mitral valve leaflets demonstrate mechanical nonlinearity and anisotropy, with a stress–strain curve that begins with a low-slope ‘toe region’, then transitions gradually to another linear region with a higher slope. These studies have also indicated that mitral valve leaflets are inhomogeneous, changing their mechanical properties going from anterior leaflet belly to the edge.^21^ However, specific measurements of certain mechanical properties such as maximum physiological radial strain values experienced by mitral valves have often varied between measurement technique and tissue source, ranging between approximately 15–25% strain.^8,19^

The maximum observed strain levels found in 3D printed samples of leaflets compared favorably with the ultimate strain values found in the porcine anterior and posterior leaflet samples measured in our study and well exceeded the maximum physiological strain values reported in these previous external studies. The architecture of sample groups ‘Leaflets C’ and ‘Leaflets D’ mimicked that of native leaflets, and the ‘Leaflets D’ group most closely matched the ultimate strain of the native leaflets. We also observed that the elastic moduli and ultimate stress measurements of printed leaflets were lower than those of porcine leaflets; however, this discrepancy was most exacerbated at higher, non-physiological strains. Ultimate force measurements of printed leaflets indicate that if a physiological force response is required at larger strains, samples with geometries larger than those of native tissue will serve the same functional purpose. Ultimately, especially at strains characteristic of maximum physiological strains, printed leaflets were able to demonstrate approximately similar stress and force response to biological leaflet tissue despite the dissimilarity in the shape of the stress–strain curve between printed materials and biological leaflets.

Printed chordae were able to best match the stress–strain response of biological chordae at low strains, corresponding to the toe region of biological chordae. There are a series of studies on the mechanical properties of porcine and human chordae, but limited information on the ranges of strains experienced by mitral valve chordae under physiological pressures. Ritchie and colleagues performed experiments with porcine chordae under physiological pressure values and found a maximum strain of 4.29 ± 3.43% throughout the cardiac cycle.^18^ Lim and Boughner performed investigation of the mechanical properties of human chordae and reported that the human chordae underwent a maximum strain level of 13%, where the toe region extended up to 6% strain and the linear region was from 6 to 12%.^13^ Liao and colleagues found that chordae with different thicknesses have different stiffnesses and extensibilities. This study indicated a maximum measured strain level of 4.3% for thin chordae, and up to 20% for thicker chordae.^12^ The ultimate strain of chordae has been reported to be 22 ± 1.2% by Zuo and colleagues.^25^ However, as physiological strain levels are largely confined to within 10% strain, we investigated the elastic modulus and forces within this physiological range.

In this study, we attempted to approximate the mechanical behavior of human chordae by replicating their elastic moduli, stress–strain curves, and force–strain curves. However, the introduction of embedded reinforcements of higher stiffness did not result in nonlinearity like that seen in biological tissue; in fact, the constructs retained the nonlinearity of opposite concavity seen in the printed annulus and leaflet samples. Future studies should involve an analysis of the flexural properties of similar printed constructs to determine if the use of reinforcements allows the constructs to exhibit greater flexibility than homogeneous constructs while maintaining mechanical stiffness.

Printed chordae constructs did demonstrate approximately similar mechanical behavior to biological chordae. Statistical analysis did not indicate a significant differences in elastic modulus between the printed and biological chordae; however, this was likely a result of the large variation in mechanical behavior between replicates for the biological samples. Testing a range of chordae from various locations of various thicknesses as a single group, in addition to the base variability of biological samples, may have contributed to this outcome. The force response of the printed constructs exceeded that of the biological chordae at all strains, and their stress response fell short at higher strains. This outcome indicates that the use of patient-specific geometries with these chordae constructs will almost certainly result in a force response that would fall short of being physiologically relevant at higher strains. Therefore, to match biological forces most closely for accurate modelling of TMVR therapies, it may be best to deviate from patient-specific chordae geometries and instead use slightly larger printed chordae constructs to compensate for their comparatively low stiffness. In our preliminary studies, we were able to accurately model the TMVR implantation in the mitral valve apparatus with slightly thicker chordae.

Constructs made of Agilus material may act mechanically different if experiencing static loading vs. rapid dynamic loading. Further work performed with this class of material should thus take this rate-dependent mechanical response into account, as physiological loading rates must be properly measured and employed if an understanding of material response under physiological conditions is desired. Physiological loading rates on components of the mitral valve apparatus tend to be much higher than those used in this study. The porcine anterior mitral valve leaflet was determined to experience maximum strain rates of approximately 10/s (1000%/s),^19^ while porcine mitral valve chordae of various sizes and from various locations experienced loading strain rates of approximately 0.7/s (70%/s, with error bar ranges between 20 and 130%/s).^18^ These values stand in contrast to the strain rate used in this study for printed materials, which was around 0.007/s (0.7%/s). It is thus possible that Agilus constructs may demonstrate higher stiffnesses that those reported in this study when deployed under such conditions.

## Conclusions

The use of 3D printed materials capable of replicating patient-specific geometries while also demonstrating biologically relevant mechanical properties could significantly improve the design and testing pipeline for medical devices, particularly the medical devices that are deployed in vivo. In this study, we demonstrate that:

(i) The photopolymers Agilus and Rigur can approximate the mechanical properties of components of the mitral valve at physiological strain levels.
(ii) That composite structures made of combinations of these photopolymers printed at different stiffnesses can recapitulate broad structural characteristics of these components, resulting in 3D printed mimics of mitral valve annuli, leaflets, and chordae.
(iii) That composites made of Agilus and Rigur are worth further investigation for the development of robust testing platforms to study the interactions between TMVR devices and biological tissue.

## Figures and Tables

**Figure 9:**
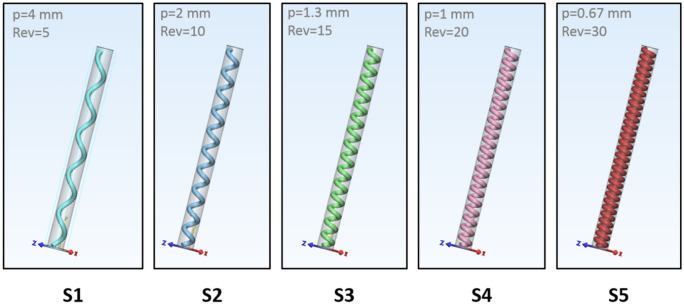
Digital 3D models of spiral-reinforced chordae with different pitches. A range of Agilus 3D-print materials were used for manufacturing chordal elements. Agilus Shore 27 was used for the base material (cylinder), and Agilus Shore 70 was used for the spiral reinforcements. Spiral diameter was set to be 1.7 mm, and reinforcement diameter was set to be 0.5 m.

**Figure 10:**
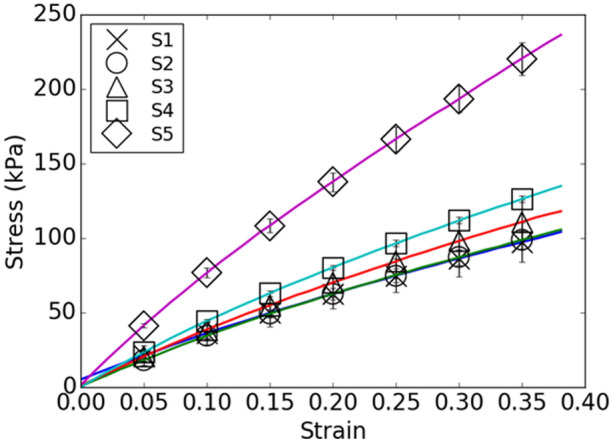
Stress–strain curves for printed chordae corresponding to 3D models. Results are presented as mean ± SD. *N *= 3 for all samples.

## Appendix

Illustration of iterative design process for printed chordae

In order to generate the finalized designs for 3D-printed constructs, a series of iterative experiments were performed for all mitral valve subcomponents discussed in this study. Here, we illustrate this process for 3D-printed chordae structures with spiral reinforcements. In the experiment described henceforth, the thickness of the reinforcement was kept constant and the pitch was modified to between 5 and 30 revolutions within a 20 mm sample length (Fig. 9).

From the stress–strain curves, it is evident that the spiral configuration with the highest pitch demonstrated the highest stiffness. Given the high stiffness of native chordae, this design was then selected for comparison against chordae with sinusoidal and rope-like reinforcements (Fig. 10).
